# Credible Complementary and Alternative Medicine Websites

**Published:** 2013-03-01

**Authors:** Jane T Williams

**Affiliations:** Jane T. Williams, MSN, RN, FNP-BC, is an advanced practice nurse at MD Anderson Cancer Center in Houston

It seems like both patients and health-care providers are constantly bombarded with information about complementary and alternative treatments on the Internet; in health and fitness magazines; on television talk shows; and from friends, family, and colleagues who are often well-intended. Most promise us better health, more energy, effortless weight loss, and cures for diseases.

In 2007, the National Health Interview Survey estimated that 38% of adults and 12% of children use complementary and alternative medicine (CAM). The Pew Internet & American Life Tracking surveys from March 2011 to February 2012 estimated that 80% of adults aged 18 to 65+ use the Internet for health and medical information. Advanced practitioners in oncology are in a prime position to help their patients and family members sort out which information is legitimate and which is misleading and possibly dangerous.

## Reliable Sources of Information

But how can advanced practitioners distinguish what is true from what is not, and how should they advise their patients? As a rule of thumb, credible websites include those ending with ".gov" (indicates a government agency), ".org" (indicates a professional and/or nonprofit organization), and ".edu" (indicates an educational institution). Beware of sites ending in ".com" as it indicates a for-profit commercial site. Here is a list of reliable CAM websites that offer evidence-based reviews of complementary therapies:

**Natural Medicines Comprehensive Database **(naturalmedicinesdatabase.com). Provides the largest number of evidence-based reviews. Authors are primarily doctors of pharmacy. Includes scientific names, uses, safety, effectiveness, mechanism of action, adverse reactions, interactions, and dosage.

**Cochrane Review Organization** (www.cochrane.org). Provides systematic reviews of therapies, including massage, acupuncture, and chiropractic interventions. Includes searches of multiple bibliographic databases by librarians.

**Natural Standard** (www.naturalstandard.com). A multidisciplinary, multi-institutional initiative for review of complementary and alternative therapies. Similar process to Cochrane reviews, with an additional historic and folkloric perspective.

**National Center for Complementary and Alternative Medicine **(nccam.nih.gov). The US government’s lead agency for scientific research on CAM. The NCCAM’s mission is to define, through rigorous scientific investigation, the usefulness and safety of CAM interventions and their roles in improving health and health care. Includes review of scientific evidence for usefulness, toxicities and precautions.

**National Cancer Institute (NCI) Office of Cancer Complementary and Alternative Medicine** (www.cancer.gov/cam). PDQ cancer information summaries specific to either patients or health-care providers. Includes background; proposed mechanisms of action; and laboratory, animal, and clinical studies.

**Memorial Sloan-Kettering Cancer Center** (www.mskcc.org/aboutherbs). Led by an oncology-trained pharmacist and a botanical expert. 

**American Cancer Society** (www.cancer.org). Provides guidelines for nutrition and physical activity for prevention, during cancer treatment, and after treatment.

**Bandolier** (http://www.medicine.ox.ac.uk/bandolier/index.html). Monthly journal about evidence-based health care produced by scientists at Oxford University. Provides a subset of analyses, commentaries, and meta-analyses of complementary therapies found in Cochrane or PubMed searches.

**The University of Texas MD Anderson Cancer Center** (www.mdanderson.org/cimer). Provides assessments of the background and evidence for complementary/integrative medicine. Also provides purchased summaries of reviews by Natural Medicines Comprehensive Database and the Cochrane Library, as well as access to reviews by the NCI and Memorial Sloan-Kettering Cancer Center.

## Let the Buyer Beware

Unfortunately, cancer patients are vulnerable to advertising, often due to fears related to their diagnosis and possibility of recurrence, and are perceived as easy targets for marketers intent on making a profit any way possible. Almost everyone has heard the advice, "If it sounds too good to be true, it probably is." Here are some common marketing tactics that patients should view with skepticism:

**Catchphrases:** Beware of language such as "scientific breakthrough," "secret ingredient," or "medical miracle"

**Conspiracy theories: **"Doctors don’t want you to know about this because it will put them out of business"

**Cure-alls: **No single treatment will cure multiple conditions or treat all cancers

**Money-back guarantees:** The marketer may be out of business before you can get a refund

**"Buy now!" promotions:** "Supplies are limited" or "Don’t miss this one-time offer"

**Technical jargon: **Sophisticated language that will obscure the fact that there is no scientific backing

## Research Claims

Oftentimes, a company will promote its product as having been evaluated through vague-sounding "research." But when these claims are investigated further, the supposed research is found to be biased, otherwise flawed, or nonexistent. Table 1 provides some guidelines for assessing the reliability of a so-called research study.

**Table 1:**
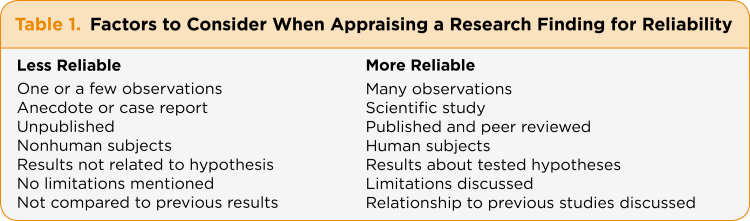
Table 1. Factors to Consider When Appraising a Research Finding for Reliability

## Final Concern

One more caveat: Many products that are promoted as "natural" may actually be made from natural ingredients but can still have detrimental side effects and may interfere with cancer treatments. Advanced practitioners in oncology should encourage their patients to report any supplements or herbal therapies that they may be taking to their health-care team.

